# Psychometric properties of the European Portuguese version of the Perceived Risk of HIV Scale in the general population and HIV-uninfected partners from sero-different couples

**DOI:** 10.1186/s12889-019-7696-y

**Published:** 2019-10-22

**Authors:** Alexandra Martins, Catarina Chaves, Maria Cristina Canavarro, Marco Pereira

**Affiliations:** Center for Research in Neuropsychology and Cognitive-Behavioral Intervention (CINEICC), Faculty of Psychology and Education Sciences of the University of Coimbra, Rua do Colégio Novo, 3000-115 Coimbra, Portugal

**Keywords:** Perceived Risk of HIV Scale (PRHS), Psychometric properties, Risk behaviours, HIV testing, HIV-uninfected partners from sero-different couples

## Abstract

**Background:**

Perceived risk of HIV plays an important role in the adoption of protective behaviours and HIV testing. However, few studies have used multiple-item measures to assess this construct. The Perceived Risk of HIV Scale (PRHS) is an 8-item measure that assesses how people think and feel about their risk of HIV infection. This cross-sectional study aimed to assess the psychometric properties (reliability and validity) of the European Portuguese version of the PRHS, including the ability of this scale to discriminate between individuals from the general population and HIV-uninfected partners from sero-different couples on their perceived risk of HIV infection (known-groups validity).

**Methods:**

This study included 917 individuals from the general population (sample 1) to assess the psychometric properties of the PRHS. To assess the known-groups validity, the sample comprised 445 participants from the general population who were in an intimate relationship (sub-set of sample 1) and 42 HIV-uninfected partners from sero-different couples (sample 2). All participants filled out a set of questionnaires, which included a self-reported questionnaire on sociodemographic information, sexual behaviours, HIV testing and the PRHS. Sample 1 also completed the HIV Knowledge Questionnaire – 18-item version.

**Results:**

The original unidimensional structure was reproduced both in exploratory and confirmatory factor analyses, and the PRHS demonstrated good reliability (α = .78; composite reliability = .82). The differential item functioning analyses indicated that the items of the PRHS, in general, did not function differently for men and women or according to HIV testing. Significant associations with sexual risk behaviours and HIV testing provided evidence for criterion validity. The known-groups validity was supported.

**Conclusions:**

The PRHS is a suitable scale in the evaluation of the perceived risk of HIV, and its psychometric characteristics validate its use in the Portuguese population. Furthermore, the present study suggests that interventions improving individuals’ HIV risk perceptions may be important since they were associated with different sexual behaviours and the likelihood of HIV testing.

## Background

At the end of 2017, there were approximately 36.9 million people living with HIV worldwide, with 1.8 million people becoming newly infected [1]. In the European Union, Portugal continues to present one of the highest incidence rates of newly diagnosed cases of HIV, despite the significant decrease of this number during the last decade. In Portugal, recent data indicate that most HIV infections continue to be acquired through HIV exposure during heterosexual sex with individuals living with HIV (60.6%) [2]. Accordingly, the identification of key components of the prevention and treatment of HIV infection and other sexually transmitted diseases (STD) to achieve the Joint United Nations Programme on HIV/AIDS (UNAIDS) targets for 2020 is of utmost importance. HIV-related knowledge is assumed to be an important component of behavioural change decision-making, although the component by itself has been demonstrated to be insufficient. Despite adequate knowledge of the disease, some literature has documented low risk perception even in the context of behaviours suggesting elevated risks for HIV exposure (e.g.,[3]).

The risk perception of HIV infection seems to be among the key factors in the adoption of safer sexual practices and HIV testing, which are crucial variables for the achievement of an HIV/AIDS-free generation, as incorrect risk perceptions may hinder efforts to diagnose and treat affected individuals [4]. The concept of risk perception has been defined as the self-reported likelihood, probability, or chance of becoming infected with HIV (e.g.,[5]). In turn, Napper, Fisher and Reynolds [6] proposed the following broader conceptualization, including different approaches, in the assessment of risk perception of HIV: (a) the cognitive assessment of risk (e.g., the likelihood of contracting HIV), (b) the intuitive assessments or feelings about risk (e.g., feeling vulnerable or worrying), and (c) the salience of the risk or how often people think about the risk (e.g., have thought about risk or can picture it happening). Regardless of its operationalization, the perceived risk of HIV has been considered an important factor to be analysed and included in HIV prevention programs [7]. In fact, according to several theories of health behaviour change, HIV risk perception is critical when motivating individuals to engage in protective behaviours, which may reduce their actual risk of infection [6]. For instance, the AIDS risk reduction model [8] has emphasized the perceived risk of HIV as a necessary path to behavioural change.

HIV risk perception has been associated with individuals’ protective (vs. risk) behaviours, including HIV testing (e.g.,[9, 10]). According to those theories of health behaviour, we might assume that if individuals perceived their risk of HIV to be high, they would be more likely to adopt protective behaviours as well as to be tested for HIV than would those who did not perceive their risk to be high [11]. In line with this assumption, the literature has documented that perceived risk of HIV and HIV testing are associated across different populations and contexts [9]. Regarding sexual behaviours, despite a diversity of results has been found, evidence has also shown a link between risk perception of HIV and these behaviours. For instance, studies have shown that a greater number of sexual partners [4, 6, 12–15], trading sex for money or drugs [6] and a younger age at first sexual intercourse [13, 14] are associated with a higher risk perception of HIV. Also, an association between condomless sex/inconsistent condom use and both the lack and higher levels of perceived risk of HIV has been suggested [6, 12, 13, 16, 17].

Several authors (e.g.,[9, 11, 18]) have argued that mixed results may be explained, at least in part, by the inconsistent measurement of the perceived risk of HIV. First, most items/questions have been formulated for each study (according to the definition adopted), thereby measuring the concept in several different ways [9]. Second, most research has used single-item measures (e.g., “What are the chances that you might get HIV?” [19]), focusing mainly on the likelihood or the cognitive assessment of risk (e.g.,[10, 12, 13, 20–23]). However, a single item may not adequately capture how people think and feel about their risk of becoming infected with HIV [6]. While some studies have suggested that perceived likelihood and affective assessments (e.g., measures focused on how often the participant worries about HIV) may be measured as separate constructs [24, 25], others have included these items in the same multiple-item scale (e.g.,[26]). Third, little research has applied multiple-item measures, specifically identifying different aspects of risk perception and reporting detailed psychometric properties of the measures employed, other than reliability (e.g.,[16, 26, 27]). Lastly, the different temporal framings used to assess the perceived risk or the use (or not) of cues or anchors in the question/item (known as the anchoring effect) may also compromise the interpretation of the findings and the comparability across studies [16]. For example, some studies have analysed the future risk perception of HIV (e.g., in the next 6 months [28]), others the current risk (e.g.,[5]), and in others it was not clear (e.g.,[14]). Additionally, several investigations have indicated that the risk assessment was based on anchors such as individuals’ sexual or alcohol/drug-related behaviours (e.g.,[15, 29]).

Filling previous gaps in the literature, Napper et al. [6] developed a comprehensive measure of the perceived risk of HIV infection, the Perceived Risk of HIV Scale (PRHS). This is an 8-item self-report measure (after an initial 10-item version) that was developed to assess how people think and feel about their risk of HIV infection based on their previous sexual behaviours. The scale is a short measure and is easy and quick to complete, which incorporates three types of items that assess different aspects of risk perception as proposed by the authors, namely, the cognitive assessments of risk, the affective or intuitive assessments, and the salience of risk. This scale has also the advantage of being adapted to analyse whether the perceived risk predicts a future behaviour by making the items conditional on a specific behaviour, such as, for example, using or not using condoms, rather than based on past sexual behaviours [6] (an item adaptation could be, e.g., “I will use condoms, if I worry about getting infected with HIV …” followed by the appropriate response options).

In the original study of the PRHS, which involved participants who were recruited from HIV testing and prevention services in Long Beach, California, the results showed that the different aspects of perceived risk loaded on a single factor and were included in a one-dimensional scale, which is contrary to what some studies had pointed out previously, suggesting that perceived likelihood and affective assessments (e.g., worry) may be independent constructs (e.g.,[25]). In the original psychometric studies, the PRHS showed excellent internal consistency (Cronbach’s alpha: .88). Evidence for convergent and criterion validity was also found, this is, the total score of the PRHS and a single-item measure of the perceived risk of HIV were strongly positively correlated; and the PRHS was also associated with measures of sexual risk behaviours (e.g., the number of sex partners). Furthermore, the scores of the PRHS differed by HIV test results (i.e., the individuals who tested positive for HIV also perceived themselves to be at higher risk), which was revealed to be a major strength of this instrument.

To our knowledge, evidence of known-groups validity of the PRHS (i.e., the ability of a measure to differentiate between groups known to differ on a certain variable) was not examined. However, some groups may perceive a higher risk of be infected with HIV than others. That may be the case of the HIV-uninfected partners in sero-different relationships (i.e., when one member of the couple lives with HIV and the other does not), who may be at substantial risk of HIV transmission (e.g.,[30, 31]). For example, Dunkle et al. [32] estimated that 55 to 93% of new heterosexually acquired HIV infections occurred within sero-different marital or cohabitation relationships in urban Zambia and Rwanda. In Portugal, because 97.5% of the HIV infections were sexually transmitted [2], it is likely that the same may also apply for most cases of HIV infection. Thus, in this population, but depending on prevention practices (not) used by sero-different couples (e.g., condom use; uptake of antiretroviral therapy [ART] by the partner living with HIV), an increased risk perception of HIV-uninfected partners of contracting HIV might be expected. For instance, previous research has found that among HIV-uninfected partners in sero-different relationships (of the Partners PrEP [pre-exposure prophylaxis] Study) the lack of condom use with the partner living with HIV, in the previous 30 days, was associated with a higher perceived risk of HIV [17].

### The present study

Despite the recent development of the PRHS, this measure has been used in research, particularly in sub-Saharan African countries [33, 34]. However, validation studies in different cultures have not been conducted. Validated and reliable instruments that measure the perceived risk of HIV are of extreme importance because they allow valuable research about how people think and feel about risk, how perceived risk relates to actual behaviours, including HIV testing, and how effective interventions that aim to reduce risk behaviours are at enhancing perceived risk [6]. Thus, the objective of this study was to examine the psychometric properties of the European Portuguese version of the PRHS in a sample of individuals from the general population, including its known-groups validity, considering a sample of HIV-uninfected partners from sero-different couples.

Specifically, the objectives of this study were (a) to explore the factor structure of the PRHS through exploratory and confirmatory analyses, (b) to investigate the likelihood of the individual items of the PRHS perform differently for two different groups (i.e., males vs. females; and individuals who had a past HIV test vs. those who had not) using differential item functioning (DIF) analyses; (c) to examine the scale’s reliability, and (d) to analyse the association between the PRHS and past sexual risk behaviours, previous HIV testing and HIV-related knowledge, which were assessed concomitantly. Additionally, this study extended the psychometric properties of the PRHS and aimed to estimate the known-groups validity, that is, the ability of the PRHS to differentiate between individuals from the general population who were in intimate relationships and the HIV-uninfected partners from sero-different couples on their perceived risk of HIV.

## Methods

### Participants and procedure

This study included both individuals from the general population as well as HIV-uninfected partners from sero-different couples. To recruit the participants from the general population, ethical approval was obtained from the Ethics Board of the Faculty of Psychology and Education Sciences of the University of Coimbra. The participants in sero-different relationships were collected in the context of a larger research project about HIV serodiscordancy. This project was approved by the Research Ethics Committees of the host institution, the National Commission of Data Protection, and three Portuguese public urban hospitals located in different cities of the country (Centro Hospitalar e Universitário de Coimbra, EPE [CHUC; Coimbra]; Hospital de Santa Maria – Centro Hospitalar de Lisboa Norte, EPE [HSM-CHLN; Lisboa]; Hospital Garcia de Orta, EPE [HGO; Almada]). None of the participants were not paid or given other incentives to participate in the study.

#### General population

In the first part of this cross-sectional study, with the main aim of assessing the factor structure of the PRHS, the sample consisted of 917 individuals (251 men, 662 women and four transsexuals [sample 1]) who were recruited online between December 2015 and December 2017 from the general Portuguese population using a convenience sampling method. Inclusion criteria were as follows: (1) being 18 years of age or older, (2) having the ability to read and understand Portuguese, and (3) not being diagnosed with HIV. Two individuals who reported being less than 18 years old and four individuals who reported that they were previously diagnosed with HIV were excluded from the study. In the second part of the study, which aimed to assess the known-groups validity, a sub-set of this sample 1 was used, and consisted of 445 participants who were in intimate relationships (94 men, 349 women and two transsexuals [group of the general population of sample 2]). This group was selected based on their relationship status (being in an intimate relationship, i.e., married, in a de facto union or in a relationship without living together) and sexual orientation (self-identified as heterosexual), to be similar to the group of HIV-uninfected partners from sero-different couples. A total of 152 married participants, 103 who were in a de facto union and 190 who were in a relationship without living together were included in this study.

The data were collected through an online survey (LimeSurvey®) that was placed on the website of the host institution, which presented information about the study aims and the ethical considerations regarding confidentiality and anonymity on the introductory page. The link to the survey was advertised through periodic online posts on the Facebook® page of the research project (both through unpaid cross-posting and through paid boosting campaigns) and was also promoted by e-mail by the host institution to the mailing lists.

#### HIV-uninfected partners from sero-different couples

The sample of the HIV-uninfected partners from Portuguese sero-different couples included 42 individuals (9 men and 33 women [group of HIV-uninfected partners of sample 2]). The participants were recruited by convenience in the departments of infectious diseases of the three Portuguese public hospitals mentioned above between September 2017 and January 2019. At the recruitment sites, both partners from a sero-different relationship were invited to participate as a couple; however, if only one member of the dyad (the one living with HIV or the other) wanted to participate, his or her sole participation was considered valid. Couples were eligible if (1) one partner was living with HIV and the other was not, (2) the partner living with HIV had disclosed his/her status to the HIV-uninfected partner, (3) they were 18 years old or older, (4) they self-defined as heterosexual individuals or as bisexual as long as the primary relationship was with a person of the opposite sex, (5) they had the ability (both language and cognitive) to complete the set of questionnaires, and (6) they provide written informed consent. Given the aims of this research project, sero-concordant couples (when both members of the couple are living with HIV), same-sex couples, as well as couples with a pregnant woman upon inclusion and if the woman (whether living with HIV or not) became pregnant during the study, were not eligible.

In the outpatient service of two hospitals (CHUC and HSM-CHLN), the infectious disease specialist briefly presented the study and asked the patient (or the couple) whether they could be contacted by the researchers after their medical consultation. If the answer was positive, the researchers, in an office provided for this purpose, presented a detailed explanation of the research objectives, explained the participants’ role and the researchers’ obligation, ensured the confidentiality of personal data, and obtained the informed consent from couples or partners who accepted to participate. The participants received an envelope containing two versions of the set of self-report questionnaires (one for the partner living with HIV and the other for the HIV-uninfected partner) to be completed at home, letters informing the participants about the study, consent forms (one for each partner), and a stamped self-addressed envelope to return the questionnaires by mail. When direct contact with one of the partners was not possible, the researchers presented the study to the partner who was present, requesting him/her to present the information received to their partner by using the letter and the informed consent (that could be returned signed later, along with the completed protocols). The couples (when both members accepted to participate) were asked to complete the assessment protocols independently. At the other hospital (HGO), the sero-different couples were identified by the infectious disease specialists, who provided the contact information (with the individuals’ consent) to the researchers, who then contacted the couples or partners and agreed upon a day to meet at the hospital to present the research and obtain the consent forms (this was preferably on a day in which the patient had to return to the hospital, thereby avoiding any additional costs).

A total of 255 couples was initially contacted, of which 209 were eligible and accepted to participate in the study. Twenty-one couples did not meet the inclusion criteria, and 28 couples refused to participate. As of January 2019, 53 sero-different couples and 15 partners in a sero-different relationship (11 living with HIV and 4 HIV-uninfected) had returned the set of questionnaires (response rate: 28.94%). Considering the aim of this study, only the data from the HIV-uninfected partners who answered the PRHS were used (*n* = 42).

### Measures

#### Sociodemographic information, sexual behaviours, and HIV testing

This information was collected through a self-report questionnaire that was developed by the researchers purposely for this study. The first part regarding the sociodemographic information was answered by all participants and asked about, for example, sex, age, marital status, relationship length and education. The second part was answered by individuals of sample 1, and included questions about past sexual behaviours and previous HIV testing. Regarding sexual behaviours, participants were asked to report their age at their first time of sexual intercourse, their number of sexual partners (during their lifetime and in the past 12 months), if they had had sex in the past 3 months (yes/no) and the respective number of sexual partners in this period (a regular partner vs. one or more occasional partners), whether they had had sex with a condom and condomless sex in the previous 3 months (yes/no) and the respective number of sexual partners with whom they had sex with a condom and condomless sex in this time period (a regular partner vs. one or more occasional partners), if they had participated in group sex in the past 3 months (yes/no) and whether they had ever received money or drugs for having sex (yes/no). Concerning HIV testing, individuals were asked whether they had ever been tested for HIV (yes/no). If the answer was positive, they were also inquired about whether they had been tested for HIV in the last year. The group of partners in sero-different couples of sample 2 were also asked to report the frequency of sex with a condom with the partner living with HIV in the past 3 months (always/never/once/two to ten times/11 to 20 times/more than 20 times).

#### Perceived Risk of HIV Scale (PRHS) [6]

The PRHS is a self-report measure that assesses three different aspects of the perceived risk of HIV: the cognitive assessments of risk (three items, e.g., “There is a chance, no matter how small, I could get HIV”), the affective or intuitive assessments (three items, e.g., “I worry about getting infected with HIV”), and the salience of risk (two items, e.g., “Picturing self getting HIV is something I find:” *Very hard to do* to *Very easy to do*). The answer scale varies from four to six options, depending on the questions. The total scores can range from 8 to 43. The total score is calculated by adding the score of each item (with item 4 being reversed), with higher scores indicating a greater risk perception.

After getting permission from the original authors to validate the PHRS, two Portuguese researchers independently translated the items to European Portuguese. The two translated versions were thoroughly compared, and a preliminary version of the PHRS was obtained. Then, a third independent researcher, who was fluent in English and not familiarized with the PRHS, translated the preliminary version back into English. The original and back-translated versions were then compared, and the translation differences were examined till the researchers reached a consensus. From this process, a measure semantically equivalent to the original PHRS was obtained. In sample 2, the Cronbach’s alpha was .76 in the group of HIV-uninfected partners and .77 in the selected participants of the general population.

#### HIV Knowledge Questionnaire – 18-item version (HIV-KQ-18) [35]

The HIV-KQ-18 is a brief self-administered measure of the 45-item HIV Knowledge Questionnaire (HIV-KQ-45) [36]. This brief version is composed of 18 items that assess the individual’s HIV-related knowledge, particularly their knowledge related to sexual transmission (e.g., “There is a female condom that can help decrease a woman’s chance of getting HIV”), using a dichotomous scale with *True*/*False* answers (the Portuguese version does not include the response option of *I don’t know*). A single overall summary score is yielded through the sum of the correct answers, with higher scores indicating a greater HIV-related knowledge. The HIV-KQ-18 was only answered by participants of sample 1.

### Data analyses

Statistical analyses were conducted using the Statistical Package for Social Sciences (SPSS), version 22.0 (IBM Corp., Armonk, NY). Confirmatory factor analysis (CFA) was performed using the Analysis of Moment Structure (AMOS), version 22.0 (IBM Corp., Armonk, NY). The Winsteps software (version 3.69.1.6) [37] was used to conduct the analyses regarding DIF.

Descriptive analyses were conducted to characterize the participants of both samples in terms of their sociodemographic, sexual behaviours and HIV testing variables. To conduct exploratory and confirmatory analyses, sample 1 was randomly divided into two halves (subsample 1: *n* = 459 and subsample 2: *n* = 458). In subsample 1, an exploratory factor analysis (EFA) with a varimax rotation was conducted to identify the factor structure of the PRHS. The Kaiser-Meyer-Olkin (KMO) test and Bartlett’s test of sphericity were conducted to test the sample’s adequacy to perform this analysis. In subsample 2, a CFA was performed to further corroborate the obtained factor structure. The method of estimation was the maximum likelihood. We examined the chi-square index (χ^2^), which indicates whether the covariation pattern in the data can be explained by the postulated factor structure. The assessment of fit was based on four additional indices: the comparative fit index (CFI), the Tucker Lewis index (TLI), the standardized root-mean-square residual (SRMR) and the root-mean-square error of approximation (RMSEA; 90% confidence interval [CI]). The model was considered to have a good fit when CFI/TLI ≥ .95, SRMR ≤ .08 and RMSEA ≤ .06 [38, 39] and an acceptable fit when CFI/TLI > .90, SRMR < .10 and RMSEA < .08 [40]. Additionally, we reported the relative chi-square (χ^2^/*df*), with ≤2 and ≤ 5 suggesting a good and an acceptable fit, respectively [41].

To ensure that the items of the PHRS work in the same way irrespective of person characteristics, DIF was investigated for sex and HIV testing. DIF analysis is particularly useful for identifying potentially biased items in measurements that may influence the total score of a questionnaire, and was evaluated by applying the Mantel-Haenszel (MH) approach using the criteria derived from the educational testing service [42]. First, the MH delta (∆_MH_) was computed by multiplying the difference in item location estimates between the groups by − 2.35 [43]. Based on the proposal of Zwick and Ercikan [44], the DIF was classified as follows: negligible (A) if |∆_MH_| was less than 1 and *p* < .05, moderate (B) if |∆_MH_| was between 1 and 1.5 and *p* < .05, and large (C) if |∆_MH_| was greater than 1.5 and *p* < .05. The DIF was also considered to be substantial if there was one absolute difference greater than 0.5 logit and a statistical significance between the difficulty parameters of the reference and the focal groups [45].

The items of the PRHS were tested regarding their mean deviation from the scale midpoint (i.e., one-sample *t*-tests, item 1: test value = 3, items 2 and 4 to 7: test value = 3.5, items 3 and 8: test value = 2.5). The reliability of the PRHS was assessed using the Cronbach’s alpha coefficient (an alpha above .70 is considered substantial [46]) and the composite reliability (CR) values, which are calculated from the squared sum of the standardized factor loading divided by the squared sum of the standardized factor loading and the error variance terms. Good reliability was established if the composite reliability was higher than .70 [47]. The corrected item-total correlations were explored and considered adequate when *r* ≥ .30. Pearson correlations were computed to assess the associations between the PRHS and the measures of sexual risk behaviours, HIV testing and HIV-related knowledge. Prior to this analysis, four variables with four and five categories (as presented in Table 2) were dichotomized (i.e., number of sexual partners in the past 12 months: 0 = none or regular partner, 1 = one or more occasional partners; number of sexual partners in the past 3 months, number of sexual partners with whom the participant had sex with a condom in the past 3 months, and number of sexual partners with whom the participant had condomless sex in the past 3 months: 0 = regular partner, 1 = one or more occasional partners).

Chi-square tests and Mann-Whitney *U* tests were performed to compare the two groups of the sample 2 on categorical and continuous variables, respectively. The known-groups validity was examined by determining the significant differences between the groups in the median PRHS total score. The Mann-Whitney *U* tests (also conducted in this analysis) were chosen considering the unequal sample sizes of the groups and the smaller sample size of the group of HIV-uninfected partners from sero-different couples. Effect sizes were calculated with Cramer’s *V* for chi-square tests and *r* for Mann-Whitney *U* test (small effects: Cramer’s *V* ≥ .01, *r* ≥ .10; medium effects: Cramer’s *V* ≥ .03, *r* ≥ .30; large effects: Cramer’s *V* ≥ .05, *r* ≥ .50 [48]). All tests were two-tailed, and a *p* value <.05 was defined as the cut-off of statistical significance.

## Results

### Results on sample 1

#### Participants’ characteristics

The sample 1 consisted of 917 participants (72.2% female) who were between the ages of 18 and 72 years old (*M* = 29.47 years; *SD* = 10.19). Most participants identified themselves as heterosexual (91.4%) and many reported being single (45.4%), although a significant proportion (51.0%) of the participants reported to be in an intimate relationship (i.e., were married, in a de facto union or in a relationship without living together); most participants also reported that they had completed university studies (82.6%), were employed (55.5%), and lived in urban areas (68.0%). Participants reported initiating sexual activity on average at the age of 17.25 (*SD* = 4.51; range: 12–32 years). Most participants reported having had one sexual partner (their regular partner) in the past 12 months (71.0%) and in the past 3 months (86.9%). Most participants also reported having had sex with a condom and condomless sex in the past 3 months with one sexual partner, specifically, their regular partner (80.1 and 91.3%, respectively). Most individuals did not participate in group sex in the previous 3 months (99.5%), nor had they ever received money/drugs for having sex (98.5%). Approximately half of the participants (50.8%) reported having been tested for HIV, although 62.9% had not been tested for HIV in the last year. The sociodemographic characteristics and sexual and HIV testing behaviours of the sample 1 and for subsamples 1 (*n* = 459) and 2 (*n* = 458) are displayed in Tables 1 and 2, respectively.

**Table 1:** Sociodemographic characteristics of the two subsamples from sample 1

	Subsample 1 (*n* = 459)	Subsample 2 (*n* = 458)	Total sample 1 (*N* = 917)
Age (years), *M* (*SD*)	29.03 (9.77)	29.91 (10.58)	29.47 (10.19)
Sex, *n* (%)			
Male	112 (24.4)	139 (30.3)	251 (27.4)
Female	344 (74.9)	318 (69.4)	662 (72.2)
Transsexual (male to female)	3 (0.3)	0 (0.0)	3 (0.3)
Transsexual (female to male)	0 (0.0)	1 (0.2)	1 (0.1)
Sexual orientation, *n* (%)			
Heterosexual	415 (90.4)	423 (92.4)	383 (91.4)
Homosexual	20 (4.4)	19 (4.1)	39 (4.3)
Bisexual	20 (4.4)	13 (2.8)	33 (3.6)
Other	4 (0.9)	3 (0.7)	7 (0.8)
Relationship status, *n* (%)			
Single	215 (46.8)	201 (43.9)	416 (45.4)
Married	75 (16.3)	83 (18.1)	158 (17.2)
De facto union	54 (11.8)	57 (12.4)	111 (12.1)
In a relationship (without cohabitating)	101 (22.0)	98 (21.4)	199 (21.7)
Divorced/Separated	13 (2.8)	17 (3.7)	30 (3.3)
Widowed	1 (0.2)	2 (0.4)	3 (0.3)
Education, *n* (%)			
Up to the 9th grade	9 (2.0)	12 (2.6)	21 (2.3)
High school (10th to 12th grade)	69 (15.0)	70 (15.3)	139 (15.2)
University studies	381 (83.0)	376 (82.1)	757 (82.6)
Work situation, *n* (%)			
Employed	240 (52.3)	269 (58.7)	509 (55.5)
Unemployed	49 (10.7)	38 (8.3)	87 (9.5)
Student	164 (35.7)	145 (31.7)	309 (33.7)
Retired	6 (1.3)	6 (1.3)	12 (1.3)
Residence, *n* (%)			
Rural area	151 (32.9)	142 (31.0)	293 (32.0)
Urban area	308 (67.1)	316 (69.0)	624 (68.0)

**Table 2:** Sexual and HIV testing behaviours of the two subsamples from sample 1

	Subsample 1 (*n* = 459)	Subsample 2 (*n* = 458)	Total sample 1 (*N* = 917)
Sexual behaviours			
Age at the first time of sexual intercourse (years), *M* (*SD*)	17.55 (4.31)^a^	16.95 (4.69)^a^	17.25 (4.51)^a^
Number of sexual partners during lifetime, *n* (%)			
None	17 (3.7)	25 (5.5)	42 (4.6)
1	123 (26.8)	113 (24.7)	236 (25.7)
2–5	215 (46.8)	207 (45.2)	422 (46.0)
> 5	104 (22.7)	113 (24.7)	217 (23.7)
Number of sexual partners in the past 12 months, *n* (%)			
None	42 (9.2)	56 (12.2)	98 (10.7)
1 – my regular partner	335 (73.0)	316 (69.0)	651 (71.0)
1 – a occasional partner	19 (4.1)	23 (5.0)	42 (4.6)
2–5	54 (11.8)	55 (12.0)	109 (11.9)
> 5	9 (2.0)	8 (1.7)	17 (1.9)
Had sex in the past 3 months, *n* (%)			
Yes	377 (82.1)	358 (78.2)	735 (80.2)
No	82 (17.9)	100 (21.8)	182 (19.8)
Number of sexual partners in the past 3 months^b^, *n* (%)			
1 – my regular partner	329 (87.3)	310 (86.6)	639 (86.9)
1 – a occasional partner	21 (5.6)	17 (3.7)	38 (5.2)
2–5	23 (6.1)	29 (6.3)	52 (7.1)
> 5	4 (1.1)	2 (0.4)	6 (0.8)
Ever had sex with a condom in the past 3 months^b^, *n* (%)			
Yes	193 (51.2)	163 (45.5)	356 (48.4)
No	184 (48.8)	196 (54.5)	379 (51.6)
Number of sexual partners with whom they had sex with a condom in the past 3 months^c^, *n* (%)			
1 – my regular partner	158 (81.9)	127 (77.9)	285 (80.1)
1 – a occasional partner	23 (11.9)	23 (14.1)	46 (12.9)
2–5	9 (4.7)	13 (8.0)	22 (6.2)
> 5	3 (1.6)	0 (0.0)	3 (0.8)
Ever had condomless sex in the past 3 months^b^, *n* (%)			
Yes	279 (74.0)	295 (82.4)	574 (78.1)
No	98 (26.0)	63 (17.6)	161 (21.9)
Number of sexual partners with whom they had condomless sex in the past 3 months^d^, *n* (%)			
1 – my regular partner	250 (89.6)	274 (92.9)	524 (91.3)
1 – a occasional partner	17 (3.7)	5 (1.7)	22 (3.8)
2–5	11 (2.4)	14 (4.7)	25 (4.4)
> 5	1 (0.2)	2 (0.7)	3 (0.5)
Participated in group sex in the past 3 months^b^, *n* (%)			
Yes	1 (0.2)	3 (0.8)	4 (0.5)
No	376 (99.7)	355 (99.2)	731 (99.5)
Ever received money/drugs for having sex, *n* (%)			
Yes	7 (1.5)	7 (1.5)	14 (1.5)
No	452 (98.5)	451 (98.5)	903 (98.5)
HIV testing			
Ever tested for HIV, *n* (%)			
Yes	230 (50.1)	236 (51.5)	466 (50.8)
No	229 (49.9)	222 (48.5)	451 (49.2)
Tested for HIV in the last year^e^, *n* (%)			
Yes	88 (38.3)	85 (36.0)	173 (37.1)
No	142 (61.7)	151 (64.0)	293 (62.9)

^a^ Number of participants that reported not to have begun their sexual life: In the subsample 1, 16 (3.5%). In the subsample 2, 23 (5.0%). In the total sample, 39 (4.3%). Range (for those who have begun their sexual life): In the subsample 1, 12–32 years. In the subsample 2, 12–30 years. In the total sample, 12–32 years

^b^ Only for those participants who had had sex in the past 3 months

^c^ Only for those participants who had had sex with a condom in the past 3 months

^d^ Only for those participants who had had condomless sex in the past 3 months

^e^ Only for those participants who had ever been tested for HIV

#### Exploratory factor analysis (EFA)

The Kaiser-Meyer-Olkin test (KMO = .86) and Bartlett’s test of sphericity [χ^2^ (28) = 1115.25, *p* < .001] confirmed the adequacy of subsample 1 (from sample 1) for the analyses. The EFA resulted in a one-factor solution (with eigenvalue > 1) that accounted for 46.0% of the total variance. The factor loadings are presented in Table 3. The items’ assignment was determined by a general rule of the factor loading exceeding .40 and having no cross-loadings.

**Table 3:** EFA (subsample 1) and CFA (subsample 2) of the PRHS

	Factor loadings in EFA	Standardized factor loadings in CFA
7. I think my chances of getting infected with HIV are	.81	.73
5. I feel vulnerable to HIV infection	.75	.68
3. Picturing self getting HIV is something I find	.72	.62
1. What is your gut feeling about how likely you are to get infected with HIV?	.70	.64
8. Getting HIV is something I have	.66	.59
4. I am sure I will NOT get infected with HIVª	.62	.42
6. There is a chance, no matter how small, I could get HIV	.57	.46
2. I worry about getting infected with HIV	.56	.62
Eigenvalue	3.68	–

*EFA* Exploratory factor analysis, *CFA* Confirmatory factor analysis

ª Reversed item

#### Confirmatory factor analysis (CFA)

The unidimensional model (previously identified by the EFA) was estimated in the second subsample (from sample 1), and the following fit indices were found: χ^2^(20) = 111.38, *p* < .001; χ^2^/*df* = 5.57; CFI = .91; TLI = .87; SRMR = .05; RMSEA = .10 (90% CI 0.08–0.12). These goodness-of-fit indices were only marginally acceptable. Consequently, the modification indices provided by AMOS were examined, which suggested that the errors belonging to items 6 and 7 might be correlated. Because the correlation of this pair of items was theoretical plausible, their measurement errors were allowed to covary [40]. The respecified model had an acceptable fit to the data [χ^2^(19) = 82.13, *p* < .001; χ^2^/*df* = 4.32; CFI = .94; TLI = .91; SRMR = .05; RMSEA = .09 (90% CI 0.07–0.11)], which was significantly better than the fit of the initial model [Δχ^2^(1) = 29.25, *p* < .001]. All standardized factor loadings for the items were significant (*p* < .001), ranging from .42 (item 4) to .73 (item 7) (see Table 3).

Because the CFA confirmed the unidimensional structure obtained in the EFA, all subsequent analyses were performed for the total sample 1 (*N* = 917).

#### Differential item functioning (DIF)

The results of the DIF analyses by sex (males vs. females) and previous HIV testing (i.e., ever tested for HIV: yes vs. no) are presented in Table 4. The data showed a minor DIF by sex and HIV testing. Only two items showed a significant DIF (i.e., contrast ≥0.50 logits and probability *p* < 0.05). One item (item 4, “I am sure I will NOT get infected with HIV”) was more difficult to endorse by females, while item 8 (“Getting HIV is something I have” Never thought about to Thought about often) was more difficult to endorse by those who had not had a previous HIV test. Considering the value of ∆_MH_, only item 8 showed a moderate DIF. The remaining items showed a negligible DIF.

**Table 4:** Differential item functioning analyses by sex and HIV testing (*N* = 917)

Item	Contrast	Welch *t*	*p*	∆_MH_	*p*
Sex					
1	0.00	0.00	1.00	0.09	.922
2	−0.08	−0.97	.332	0.31	.137
3	0.19	1.53	.127	0.19	.237
4	−0.50	6.84	< .001	0.73	< .001
5	0.29	3.57	< .001	0.38	< .001
6	0.08	1.12	.264	0.07	.180
7	0.21	1.80	.073	0.21	.224
8	−0.09	− 0.72	.471	0.38	.268
HIV testing					
1	0.32	2.95	.003	0.85	< .001
2	0.22	2.96	.003	0.09	.010
3	0.07	0.59	.553	0.71	.266
4	0.00	0.00	1.00	0.19	.648
5	0.11	1.55	.122	0.38	.042
6	−0.15	−2.28	.023	0.33	.012
7	−0.11	−1.04	.301	0.07	.830
8	−0.59	−5.43	< .001	1.27	< .001

#### Descriptive statistics and reliability

The descriptive analyses of each PRHS item are presented in Table 5. There were no missing values for any item. Most items (i.e., six items) scored significantly below the scale midpoint, meaning that the participants tended to disagree with these items. One item (item 6) scored significantly above the scale midpoint, showing that the participants tended to agree with this item. The mean of one item (item 4) did not differ significantly from the scale midpoint. No ceiling effects were observed; however, floor effects were detected in five items. The skewness and kurtosis coefficients of most items were within the acceptable range of ±1.00.

**Table 5:** Descriptive statistics and reliability of items of the PRHS (*N* = 917)

PRHS items	Range	*M* (*SD*)	Floor (%)	Ceiling (%)	S	K	Corrected item-total correlation	Cronbach’s alpha if item deleted
1. What is your gut feeling about how likely you are to get infected with HIV? (*Extremely unlikely* to *Extremely likely*) [A]	1–5	1.73^b^ (0.76)	43.5	0.7	0.94	1.21	.55	.76
2. I worry about getting infected with HIV (*None of the time* to *All of the time*) [A]	1–6	2.45^b^ (1.23)	20.1	3.1	1.05	0.68	.45	.77
3. Picturing self getting HIV is something I find: (*Very hard to do* to *Very easy to do*) [S]	1–4	1.58^b^ (0.70)	52.7	1.5	1.06	0.83	.55	.76
4. I am sure I will NOT get infected with HIV (*Strongly disagree* to *Strongly agree*) ª [C]	1–6	3.56 (1.53)	10.4	9.3	−0.13	−1.15	.44	.78
5. I feel vulnerable to HIV infection (*Strongly disagree* to *Strongly agree*) [A]	1–6	2.34^b^ (1.25)	29.3	0.9	0.77	−0.30	.61	.74
6. There is a chance, no matter how small, I could get HIV (*Strongly disagree* to *Strongly agree*) [C]	1–6	3.84^c^ (1.58)	10.9	13.3	−0.44	−0.99	.45	.78
7. I think my chances of getting infected with HIV are (*Zero* to *Very large*) [C]	1–6	2.45^b^ (0.79)	9.7	0.2	0.30	0.76	.71	.74
8. Getting HIV is something I have (*Never thought about* to *Thought about often*) [S]	1–4	2.08^b^ (0.75)	21.5	2.6	0.25	−0.34	.52	.76

Floor (%): Percentage of participants at the lowest scale rating. Ceiling (%): Percentage of participants at the highest scale rating. *S* Skewness. *K* Kurtosis. Item type: [A] = Affective item; [S] = Salience item; [C] = Cognitive item

ª Reversed item

^b^ Item mean below the scale midpoint (one-sample *t* test, *p* < .05)

^c^ Item mean above the scale midpoint (one-sample *t* test, *p* < .05)

An adequate internal consistency was found for the European Portuguese version of the PRHS (Cronbach’s alpha = .78). The composite reliability value calculated from the standardized factor loadings of the PRHS items was higher than the recommended threshold of .70 (CR = .82). The exclusion of each item did not contribute to an increase in the alpha coefficient. All corrected item-total correlations were above .40, indicating that each item was related to the overall scale (see Table 5).

#### Association with other variables

The correlations between the PRHS and the variables of sexual risk behaviours, HIV testing and HIV-related knowledge were analysed (see Table 6). The perceived risk of HIV was significantly and positively associated with the number of sexual partners in the past 12 months, the number of sexual partners in the past 3 months, the number of sexual partners with whom the participant had sex with a condom in the past 3 months, the number of sexual partners with whom the participant had condomless sex in the past 3 months, having had participated in group sex in the past 3 months, and having ever received money or drugs for having sex. However, note that for these two last variables, the number of the participants who reported an affirmative answer was very small (*n* = 4 and *n* = 14, respectively). The perceived risk of HIV was also significantly and positively associated with HIV testing, this is, with having ever been tested for HIV and having been tested in the last year. No significant correlations were found with age at the first time of sexual intercourse and HIV-related knowledge.

**Table 6:** Correlations between the PRHS and sexual behaviours, HIV testing and HIV-related knowledge (*N* = 917)

	PRHS
Age at the first time of sexual intercourse	.03
Number of sexual partners in the past 12 months	.23***
Number of sexual partners in the past 3 months	.22***
Number of sexual partners with whom the participant had sex with a condom in the past 3 months	.22***
Number of sexual partners with whom the participant had condomless sex in the past 3 months	.25***
Group sex in the past 3 months	.14***
Ever received money/drugs for having sex	.08*
Ever tested for HIV	.14***
Tested for HIV in the last year	.13**
HIV-related knowledge	.05

*p < .05; **p < .01.; ***p < .001

### Results on sample 2: known-groups validity

#### Participant’s characteristics

The sample 2 consisted of 445 participants from the general population who were in intimate relationships and were between the ages of 18 and 72 years old (*M* = 31.89 years; *SD* = 11.03) and 42 HIV-uninfected partners from sero-different couples who were between the ages of 24 and 67 years old (*M* = 47.45 years; *SD* = 11.45). Most participants were female, employed and living in urban areas. Significant differences between the groups were found regarding age, relationship length, education and professional status. While most participants from the general population had completed university studies (83.8%), the HIV-uninfected partners from sero-different couples reported having a 9th grade or below education (51.2%). On average, the participants from the general population had been in their current intimate relationship for 110.63 months (*SD* = 112.54; range: 1 month-49 years), and the HIV-uninfected partners from sero-different couples had been in their relationship for an average of 180.59 months (*SD* = 158.58; range: 5 months-47 years). Table 7 summarizes the sociodemographic characteristics and comparison of the groups. Regarding condom use by the HIV-uninfected partners, 46.3% (*n* = 19) of these partners reported that they never had sex with a condom with their partner living with HIV in the past 3 months, and 36.6% (*n* = 15) mentioned that they always had. In addition, 2.4% (*n* = 1) reported condom use only once, 7.3% (*n* = 3) two to ten times, 4.9% (*n* = 2) reported 11 to 20 times, and 2.4% (*n* = 1) more than 20 times.

**Table 7:** Sociodemographic characteristics of the groups from sample 2

	General population (*n* = 445)	HIV-uninfected partners (*n* = 42)	*χ* ^2^	Cramer’s *V*
	*n* (%)	*n* (%)		
Sex, *n* (%)			0.19	.02
Male	94 (21.1)	9 (21.4)		
Female	349 (78.4)	33 (78.6)		
Transsexual (male to female)	2 (0.5)	0 (0.0)		
Transsexual (female to male)	0 (0.0)	0 (0.0)		
Education, *n* (%)			139.59***	.54
Up to the 9th grade	15 (3.4)	21 (51.2)		
High school (10th to 12th grade)	57 (12.8)	11 (26.8)		
University studies	373 (83.8)	9 (22.0)		
Work situation, *n* (%)			41.24***	.29
Employed	284 (63.8)	21 (55.3)		
Unemployed	47 (10.6)	10 (26.3)		
Student	107 (24.0)	1 (2.6)		
Retired	7 (1.6)	6 (15.8)		
Residence, *n* (%)			0.65	.04
Rural area	147 (33.0)	15 (39.5)		
Urban area	298 (67.0)	23 (60.5)		
	*Median (IRQ)*	*Median (IRQ)*	*U*	*r*
Age (years)	28 (14)	45 (18.25)	2982.00***	.33
Relationship length (months)	69 (126.5)	140 (180)	6177.50**	.16

For the HIV-uninfected partners from sero-different couples, the *n*s of variables do not add up to 42 due to missing values. The number of missing responses in sociodemographic data ranged from 0 to 4. IRQ: Interquartile range

** *p* < .01; *** *p* < .001

#### Differences between groups on perceived risk of HIV

The HIV-uninfected partners from sero-different couples (median = 23; IRQ = 9) scored higher on the PRHS than did the individuals from the general population who were in intimate relationships (median = 19; IRQ = 7). The Mann-Whitney *U* test was found to be 5988.50 (Z = − 3.86), *p* < .001, *r* = .17, indicating significant differences between the individuals from the general population and the HIV-uninfected partners from sero-different couples on their perceived risk of HIV.

## Discussion

The objective of our study using different samples (i.e., individuals from the general population and HIV-uninfected partners from sero-different couples) was to test the psychometric properties of the European Portuguese version of the PRHS. Specifically, it aimed to analyse the factor structure of the PRHS, to investigate the DIF considering sex and HIV testing, to examine the scale’s reliability, to explore the association between the PRHS and sexual risk behaviours, HIV testing and HIV-related knowledge, and to estimate the known-groups validity of the scale.

Overall, the psychometric characteristics of the PRHS were very satisfactory, with demonstrated reliability and construct, criterion and know-groups validity. Supporting the construct validity of the PRHS and consistent with the original version of the scale, the results of the EFA suggested a single factor, which was confirmed through a CFA. The different fit indices of the model obtained in the CFA resemble those presented in the original study of the scale [6]. To improve the model fit, the errors belonging to items 6 and 7 were allowed to covary. However, the correlation of these errors was theoretical plausible because they were both cognitive items that assessed the self-reported chance of becoming infected. Additional support for the construct validity of the PRHS, specifically its known-groups validity, was found using a sample of individuals from the general population who were in intimate relationships and the HIV-uninfected partners from sero-different couples. Because these partners are in an intimate relationship with a partner living with HIV, they are also at a higher risk of HIV transmission (e.g.,[31]). Therefore, our finding indicating a higher perceived risk among the HIV-uninfected partners from sero-different couples, when in comparison with individuals from the general population, is not only expected, but it is also crucial for prevention, as it may suggest the importance of promoting protective behaviours within the relationship, such as condom use. In fact, in our group of HIV-uninfected partners, almost half of the participants (46.3%) reported never having had sex with a condom with their partner in the previous 3 months. Regardless the viral suppression of the partner living with HIV, it seems relevant to reinforce condom use, specifically, within these couples, since they may also be in a higher risk of acquiring other STDs [49].

Most items did not demonstrate a significant DIF. Overall, this suggests that PRHS items did not function differently for men and women or according to previous HIV testing, as well as that these items were not particularly biased, therefore influencing the total score of the PHRS. However, one item (item 8) presented a moderate DIF by HIV testing. This item assesses how often individuals think about getting HIV (*Never thought about* to *Thought about often*) and it was more difficult to endorse by those who had not been previously test for HIV. This result could possibly be explained by the fact that individuals who never have had the test are also those who have probably engaged in fewer risk behaviours before and who perceive themselves as being at a low risk of HIV. Although this DIF may be of little practical significance, further inspection in terms of possible item-level bias in the measurement of perceived HIV risk is warranted.

Despite a Cronbach’s alpha lower than that reported in the original study (α = .88), the values of the Cronbach’s alpha coefficient (α = .78) and the composite reliability (CR = .82) were rather acceptable [47]. The Cronbach’s alpha in the group of HIV-uninfected partners was also satisfactory, exceeding the recommended alpha of .70 [46]. At the item level, all items appeared to be worthy of retention, and the corrected correlation values between each item of the scale and the total score indicated a good discriminating power of the items. Despite no ceiling effects found, the existence of floor effects above the accepted threshold of 15% should be considered [50], particularly in two items (items 1 and 3). The characteristics of our sample 1 may have had a role in these observed floor effects in the individual items because these effects are population dependent [51]. In fact, 19.8% of the participants of that sample reported not being sexually active in the past 3 months or only having sex with their regular partner (86.9%); therefore, they may not perceive risk in their sexual behaviours. In line with this, the analysis of the mean deviation from the scale midpoint of the items of the PRHS also revealed that the participants tended to disagree with most of them.

The evidence for criterion validity of this measure was supported by the significant associations between the total score of the PRHS and the measures of sexual risk behaviours and HIV testing. Regarding sexual risk behaviours, as expected, the variables of having had one or more occasional partners in the past 12 and 3 months, including having had condomless sex with one or more occasional partners in the past 3 months, having had participated in group sex in the past 3 months and having ever received money or drugs for having sex were associated with a higher perceived risk of HIV. These associations are consistent with past literature (e.g.,[4, 6, 12]) showing that individuals who engage in risk behaviours perceive themselves as being at a higher risk of HIV infection. However, a positive association with having had sex with a condom with one or more occasional partners in the previous 3 months was also found. This seems to suggest that participants who have sex with occasional or multiple sexual partners, even while using condoms, may perceive themselves as having a higher risk of HIV infection (and the perceived risk reflects the number of partners rather than the protective behaviour, i.e., condom use). Indeed, Do and Meekers [52] found that having multiple partners was the strongest predictor of the perceived risk of HIV, independent of other confounding factors such as condom use at last sexual relationship. Nevertheless, given the cross-sectional design of the study, these associations may be bidirectional, and thus, an opposite explanation is also plausible. Corroborating prior meta-analytic findings [9], in our study, higher scores on the PRHS were also associated with both having ever been tested for HIV and having been tested for HIV in the last year.

In this study, contrary to prior findings [13, 14], the association between age at the first time of sexual intercourse and the perceived risk of HIV was nonsignificant. This outcome may be related to the sample distribution of our study, which shows little variability regarding this variable (the vast majority of participants initiated their sexual activity at the ages of 16–18). Additionally, we did not find a significant association between the perceived HIV risk and the measure assessing the participant’s HIV-related knowledge. This is an expected result, which is consistent with the findings of a recent systematic review [53] that showed that most of the studies did not find any association between HIV/AIDS-related knowledge and risk perception.

Some limitations should be considered. First, a convenience sampling method was used, which limits the generalizability of the results. For example, 82.6% of participants of sample 1 had university studies, which is not representative of the general Portuguese population. In addition, the small sample size of the HIV-uninfected partners from sero-different couples, as well as the low response rate, constrains the representativeness of this population. Therefore, inferences about our results should be interpreted with caution and within the context of these samples’ characteristics. Second, the cross-sectional design of the study did not allow us to examine the instrument’s temporal stability or responsiveness to change. The use of longitudinal designs in future studies would be helpful to overcome these limitations and to examine the complex relationship between perceived risk and behaviour (e.g., the association with condom use) [6]. Third, given the sensitive nature of many survey questions (e.g., participating in group sex), it is possible that some participants provided socially acceptable responses. However, we consider that the data collection of individuals from the general population through an online survey (despite the associated limitations regarding this recruitment method [e.g., selection bias, as the study was limited to individuals with Internet access]) may have enhanced anonymity and minimized social desirability, thereby allowing us to achieve more reliable results.

## Conclusions

Despite the previously specified limitations, this study represents an important contribution to the measurement of the risk perception of HIV, providing the validation of the PRHS in a different culture and with different populations (the general population and a group at a higher risk of HIV infection, i.e., the uninfected partners of an HIV sero-different relationship) and, to the best of our knowledge, is the first study to establish a comparison of the perceived risk of HIV infection between these groups. Overall, this study present evidence supporting the reliability and validity of the European Portuguese version of the PRHS, which is a multiple-item measure that incorporates different aspects of the perceived risk of HIV and is closely related to individuals’ previous sexual behaviours, and therefore attest its use in the assessment of perceived risk in the Portuguese population. Our results also suggest that perceived risk of HIV is a promising tool to use when trying to engage individuals in interventions focused on motiving people to protect themselves and, consequently, on how to reduce their perceived risk. In fact, the health belief model [8, 9] has identified that the perception of HIV risk, recognition of its seriousness, and knowledge about its prevention influence safer sexual activity. Relatedly, Johnston et al. [12] stated that individuals who accurately recognize themselves at risk, acknowledge their vulnerability to HIV infection and may become more receptive to HIV education and related services. Furthermore, because PRHS is a more comprehensive measure of this construct than are single-item measures (although brief), it may be useful to include it as an instrument to apply before and after such interventions, thus contributing to the assessment of its efficacy.

## Data Availability

The datasets used and/or analysed during the current study are available from the corresponding author on reasonable request (alexandrafrsmartins@gmail.com).

## Abbreviations

AIDS: Acquired immunodeficiency syndrome
AMOS: Analysis of Moment Structure
ART: Antiretroviral therapy
CFA: Confirmatory factor analysis
CFI: Comparative fit index
CHUC: Centro Hospitalar e Universitário de Coimbra
CI: Confidence interval
CR: Composite reliability
DIF: Differential item functioning
EFA: Exploratory factor analysis
HGO: Hospital Garcia de Orta
HIV: Human immunodeficiency virus
HIV-KQ-18: HIV Knowledge Questionnaire – 18-item version
HIV-KQ-45: HIV Knowledge Questionnaire – 45-item version
HSM-CHLN: Hospital de Santa Maria – Centro Hospitalar de Lisboa Norte
KMO: Kaiser-Meyer-Olkin test
MH: Mantel-Haenszel
PrEP: Pre-exposure prophylaxis
PRHS: Perceived Risk of HIV Scale
RMSEA: Root mean square error of approximation
SPSS: Statistical Package for Social Sciences
SRMR: Standardized root mean squared residual
STD: Sexually transmitted disease
TLI: Tucker Lewis index
UNAIDS: Joint United Nations Programme on HIV/AIDS
